# Design, implementation and validation of a novel open framework for agile development of mobile health applications

**DOI:** 10.1186/1475-925X-14-S2-S6

**Published:** 2015-08-13

**Authors:** Oresti Banos, Claudia Villalonga, Rafael Garcia, Alejandro Saez, Miguel Damas, Juan A Holgado-Terriza, Sungyong Lee, Hector Pomares, Ignacio Rojas

**Affiliations:** 1 Department of Computer Architecture and Computer Technology, Research Center for Information and Communications Technologies of the University of Granada (CITIC-UGR), Periodista Rafael Gomez Montero 2, E18071 Granada, Spain; 2 Department of Computer Engineering, Kyung Hee University, 1732 Deokyoungdae-ro, Giheung-ug, 446-701 Yongin-si, South Korea

**Keywords:** mHealth framework, mobile health, digital health, portable sensors, wearable sensors, biomedical sensors, health devices, activity recognition, human behavior

## Abstract

The delivery of healthcare services has experienced tremendous changes during the last years. Mobile health or mHealth is a key engine of advance in the forefront of this revolution. Although there exists a growing development of mobile health applications, there is a lack of tools specifically devised for their implementation. This work presents mHealthDroid, an open source Android implementation of a mHealth Framework designed to facilitate the rapid and easy development of mHealth and biomedical apps. The framework is particularly planned to leverage the potential of mobile devices such as smartphones or tablets, wearable sensors and portable biomedical systems. These devices are increasingly used for the monitoring and delivery of personal health care and wellbeing. The framework implements several functionalities to support resource and communication abstraction, biomedical data acquisition, health knowledge extraction, persistent data storage, adaptive visualization, system management and value-added services such as intelligent alerts, recommendations and guidelines. An exemplary application is also presented along this work to demonstrate the potential of mHealthDroid. This app is used to investigate on the analysis of human behavior, which is considered to be one of the most prominent areas in mHealth. An accurate activity recognition model is developed and successfully validated in both offline and online conditions.

## Background

Traditional processes and services for the delivery of health and care are experiencing a drastic shift to meet people present and future needs. The current proliferation of mobile technologies is helping to pave the path to a new paradigm in which people's health information is timely and ubiquitously available. Concretely, portable and wearable sensors are increasingly utilized to collect data on individuals' biology, psychology and behavior. This valuable information may be used to reduce health risks, optimize health outcomes, understand the determinants of health or even yield new insights into the factors that lead to disease.

Recent surveys show a growing tendency in physicians mobile and digital health adoption. Mainstream applications in the medical domain are principally devoted to learning and informative purposes. Examples of these apps are *Medscape *[1] and *Epocrates *[2], which are particularly intended to provide comprehensive and updated information for medical procedures, disease monographs, drug references or practice guidelines. Other applications are mostly useful for primary care practitioners or generalists, such as *Calculate by QxMD *[3], which provides them with medical calculators and decision support tools that apply to several medical specialties. Electronic reference manuals are also close at hand, as it is the case of *Monthly Prescribing Reference *[4], an app that incorporates prescribing notes and drug records which facilitate clinical practice and promote the access to the latest advances in treatments [5]. Physicians also increasingly recommend the use of health apps to patients [6]. While most of these apps require users to actively report about their health conditions, e.g., through annotating dietary habits [7] or daily routines [8], new technological trends seek to benefit from the information collected through wearable biomedical devices. Built-in motion sensors readily available in smartphones are used, for example, to detect abnormal conducts such as elderly falls [9] or freezing of gait in Parkinson's patients [10]. Other applications develop on data collected through external wearable health devices capable of inferring sleep disorders [11], detecting cardiovascular illnesses [12], alerting on physical conditions [13] or tracking changes in physiological responses of patients with chronic obstructive pulmonary disease [14].

Despite the rise of mHealth technology, this field is far from mature. Consumers' demand for health apps and sensors clearly outpaces the science needed to understand their benefits, risks and impact on health outcomes. In fact, researchers and developers still need to build and assess the complete spectrum of mHealth technologies, as they create safe, scalable and effective applications. To that end, powerful frameworks and tools that support the development and validation of multidisciplinary mHealth applications are required. There exist various attempts to this respect. For example, [15] provides an open source electrocardiogram signal processing code for quality analysis and atrial fibrillation screening. In [16] the authors present a mobile phone platform to collect users' psychological, physiological and activity information for mental health research. A mobile version of a data processing toolbox originally devised for computer-based architectures and principally used for human behavior modeling is provided in [17]. Distributed signal processing algorithms for the analysis and classification of sensor data are provided as part of a framework for rapid prototyping of body sensor networks in [18]. A mHealth middleware framework integrating multiple interfaces and multiparameter monitoring of physiological measurement is proposed in [19]. Tools to analyze the provenance of mHealth data have also been suggested in [20]. Despite the effort put on the development of health frameworks and tools, past contributions either focus on a specific domain or lack some essential features of health applications.

## Requirements of a mHealth framework

The main goal of mHealth frameworks is to foster the research and development in health and medical domains as well as to accelerate the market of mobile health technologies and applications. To that end, two supportive objectives are devised: 1) to allow developers to rapidly build applications while integrating a wide spectrum of mobile health devices; 2) to grant simple access, representation and processing of health data collected through heterogeneous resources across several applications.

During the design of a mHealth framework several requirements must be borne in mind, the essential of which are outlined in the following. A certain level of abstraction from heterogeneous resources should be ensured to make hardware and its communication transparent to the developer. For the sake of interoperability, the framework should also define a unified model for multimodal health data. Medical and health applications can operate on a local basis or remotely, thus it should be supported both local and remote storage of health data. Raw medical and physiological data is normally analyzed to extract health knowledge. Accordingly, a mHealth framework should include signal processing, data mining and machine learning techniques tailored to mHealth applications. In this regard, mechanisms to visualize medical and health information in a user-friendly fashion must be also provided for both average users and specialists. Another major requirement refers to the provision of healthcare services such as health delivery, personalized guidelines and intelligent recommendations. Finally, the framework should be modular and extensible to future sensor technologies and application needs.

## Architecture of the mHealth framework

**Figure 1:**
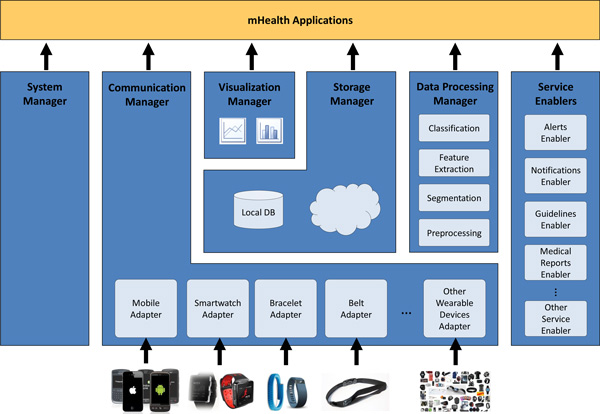
**mHealth Framework**.

In the light of the requirements presented in the previous section, a novel framework devised to enable the easy and agile development of mHealth applications leveraging on heterogeneous wearable biomedical devices is proposed. The mHealth Framework implements several functionalities to support resource and communication abstraction, biomedical data acquisition, health knowledge extraction, persistent data storage, adaptive visualization, system management and value-added services. Figure 1 shows the architecture that implements these functionalities and the components of the mHealth Framework.

In a nutshell, mHealth data delivered by mobile and biomedical sensors is collected and structured by the Communication Manager. This raw data can be stored in the Storage Manager, further processed by the Data Processing Manager, graphically represented by the Visualization Manager or directly used by the applications built on the mHeath Framework. Moreover, the medical knowledge derived by the Data Processing Manager can also be stored in the Storage Manager, input to advanced functionalities provided by the Service Enablers or used by the mHealth applications. Since the Storage Manager offers persistence, stored data can be offline processed by the Data Processing Manager, graphically represented by the Visualization Manager or accessed by the mHealth applications. Finally, the mHealth Framework offers, by means of the System Manager, functionalities to manage general resources of the mobile or wearable device.

### Communication Manager

mHealth applications may operate on multiple heterogeneous mobile and biomedical devices. The Communication Manager provides the abstraction level required to enable the functioning of applications independently of the underlying health technologies. This component removes the burden of communicating with several heterogeneous wearable devices; thus, making the communication transparent to the application and to the other framework components. Moreover, this manager serves as interpreter of the multimodal health data, providing a unified data model (see Section "Data Model") understandable by the rest of the framework components and mHealth applications.

In order to procure transparent communication and data retrieval, the Communication Manager incorporates Adapters, which are standalone modules devised to support the use of an specific mobile or biomedical device. The Adapter manages the connection with the device, interprets the received data and maps it to the unified data model. The modularity of the Adapters makes the Communication Manager extensible and evolvable to future devices and technologies.

### Storage manager

The Storage Manager provides data persistence both locally and remotely. It enables the easy retrieval of stored data, abstracting the queries from the storage system behind. This manager is also responsible for the efficient synchronization of the data and its secure transmission to the remote store, either in the cloud or remote server.

### Data processing manager

The Data Processing Manager is in charge of the processing of health data by providing signal processing, data mining and machine learning techniques. The processing functionalities can run in two different operation modes. The first mode operates online by processing the data collected at runtime by the Communication Manager. The second approach functions on an offline manner by retrieving the data from the Storage Manager. The Data Processing Manager includes four independent modules each one corresponding to the stages of the data processing chain: preprocessing, segmentation, feature extraction and classification.

#### Preprocessing

The health data collected through the sensors may be affected by diverse type of artifacts such as spurious spikes or electronic noise, or be loosely controlled resulting in abnormal values and inconsistencies. Accordingly, it may be necessary to remove these anomalies from the raw data, for example, by using filtering or screening techniques. The Preprocessing module is devised to apply mechanisms to clean, transform and ultimately adequate the data to the application needs.

#### Segmentation

Continuous biodata streams need to be split into discrete segments or pieces to be further processed. For example, sliding window approaches are commonly used for the segmentation of body-motion data. The Segmentation module provides diverse techniques to partition the data.

#### Feature extraction

The feature extraction process is performed to provide a more tractable representation of the biosignals for the pattern recognition or mining stage. The Feature Extraction module permits to transform the input data into a reduced representation set of features or feature vector. Depending on the particular application area or domain some features may be preferentially used. Examples of features are statistical functions such as the mean or median, time/frequency transformations, and heuristics, which are provided by the Feature Extraction module.

#### Classification

Artificial intelligence algorithms are widely used to gain knowledge from the collected health data. The features extracted by the Feature Extraction module are input to this type of algorithms provided by the Classification module to eventually categorize the data into a particular class or concept. The identified classes, which may represent health conditions and behavioral patterns, can be used by the Service Enablers and mHealth applications.

### Visualization manager

The data representation is a fundamental element of any mHealth app. Since applications may have different objectives and target users, developers require a wide sort of graphical representation tools. The Visualization Manager is in charge of providing diverse modes and ways to display data. This manager may operate on the data provided by the Communication Manager or the Storage Manager. An 'online' mode is identified for the depiction of the data provided by the Communication Manager, which corresponds to the information collected by the health sensors at runtime. On the other hand, an 'offline' operation mode is defined for the data saved in the permanent storage. Not only raw signals may be represented but also the information obtained after the data processing.

### System manager

The System Manager provides developers with functionalities to manage general resources of the mobile device. Examples of these resources are wireless connections (WiFi, 3G connection, Bluetooth), geopositioning technologies (GPS), screen configuration or battery management, among others.

### Service enablers

An important characteristic of several mobile health applications is the intervention on health states. Health data may be profited to influence elements of the intervention and yield new information from which to act. This information is here devised to be provided to the users through a set of Service Enablers, which support alerts, notifications, guidelines, reports and other future advanced functionalities.

#### Alerts enabler

The Alerts Enabler provides mechanisms to trigger alerts and emergency procedures when abnormalities or risk situations are detected. Examples of these mechanisms are automatic phone calls and messages, which may be delivered to the patients' family, carers and emergency services in the event of a critical situation, e.g., after detection of a fall or cardiac anomaly.

#### Notifications enabler

Users may need to be timely or occasionally informed about important facts of their healthcare and wellbeing process. Health remainders, e.g., medication intake or prescribed exercise, are essential mechanisms to engage users in the care process, to procure their organization and to empower them to meet the treatment goals. The Notifications Enabler is devised to support prescheduled or event-based user-friendly notifications that may also trigger additional services.

#### Guidelines enabler

Instructions, encouragements and educational information from specialists are of high value to promote healthy lifestyles and to support the patient self-care. The Guidelines Enabler provides multimedia tools for displaying guidelines that may be personalized and adapted to the user's needs and conditions.

#### Medical reports enabler

The Medical Reports Enabler is devised to facilitate the structuring of the medical knowledge in an expert-oriented standardized format, e.g., HL7. This enabler will help clinicians and care professionals to interpret health trends and to support medical decisions.

### Data model

A unified Data Model enables data interoperability required to ensure intercommunication among the mHealth Framework components and applications. The model has to be generic, flexible and extensible to support the representation of heterogeneous data collected by present and future mobile and wearable devices. This is of utmost importance due to the variety of sensing modalities used in mHealth. The mHealth Data Model comprises five elements. The Session object is the main element and represents a user's health data recording session, including its metadata. The Session is composed of several Sample objects which refer to each sample from the biodata stream collected during the session. Each Sample links to multiple Device objects which represent the mobile and wearable devices streaming during the session. Since a device offers different sensor modalities, the Device links to the Sensor objects. The Sensor contains the data collected by a given sensor in a specific moment. Metadata is required to interpret the data collected by the multimodal sensors and the different devices. Since the metadata does not vary during a recording session, and to reduce the model overhead, the Metadata object is associated to the Session object instead of to each Sample, Device or Sensor object. The Metadata defines the types of sensors, the units of the measurements, the start and end time of the recorded session and the sampling rate.

## mHealthDroid: an Implementation of the mHealth framework

mHealthDroid is the Android implementation of the previously proposed mHealth Framework. It is released open source under the GNU General Public License version 3 and available at [21]. mHealthDroid is devised to operate on the Android operating system version 4.2 ("Jelly Bean"), although it provides backwards compatibility to version 2.3.3 ("Gingerbread"). The managers of the mHealth Framework architecture have been developed using the singleton pattern approach in mHealthDroid.

The mHealthDroid Communication Manager has been implemented to facilitate the incorporation of new Adapters. To do so, it provides a generic Adapter skeleton. The current implementation of mHealthDroid provides the Adapter for Android mobile devices and the Adapter for the Shimmer2 and Shimmer3 wearable devices [22]. The Android Mobile Adapter abstracts the sensors embedded into the mobile device (e.g., GPS, temperature or humidity). Likewise, the Shimmer Adapters provide the means to communicate the wearable device with the mobile device and to map the data to the proprietary format. Shimmer devices provide multiple sensing modalities that span from inertial sensing via accelerometer, gyroscope, magnetometer, and altimeter, to physiological signs measurement such as electrocardiogram or electromyogram, among others.

The Storage Manager incorporates a SQLite database [23] to implement the local persistence functionality. SQLite is a popular database engine on memory constrained systems, like mobile devices, since it runs in minimal stack space and very little heap. The Storage Manager also offers an interface to easily retrieve, based on diverse identifiers (session, device identifier, date, time interval), the data stored in the SQLite database. Database consistency check procedures are implemented by the Storage Manager to ensure integrity in the synchronization between the remote and local storage. The transmission to the remote storage is implemented using a HTTP POST request method, which encloses in the request message's body the JSON [24] representation of the data. mHealthDroid also offers a server side implementation for remote persistence. This implementation builds on a MySQL [25] database and provides PHP scripts that use the MySQLi API [26] to manage the remote database.

The mHealthDroid Data Processing Manager provides an essential set of functionalities typically used in the data processing chain. The Preprocessing module implements two techniques: upsampling to increase the sampling rate and downsampling to reduce the sampling rate. A sliding window approach, widely-used in signal processing problems, is implemented by the Segmentation module. The Feature Extraction module implements some generic statistical features such as mean, variance, standard deviation, zero crossing rate, mean crossing rate, maximum and minimum. The Classification module builds on an open source stripped version [27] of Weka (Waikato Environment for Knowledge Analysis [28]). It provides functionalities to train and validate machine learning models, that can be used for classification purposes. mHealthDroid currently implements Naive Bayes [29], Adaboost [30], Decision Trees [31], Linear Regression [32] and ZeroR [33] classification techniques.

The Visualization Manager builds on the open source library Graphview [34], which has been adapted to fulfill the particular needs of mHealth data representation. The manager allows multiplot visualization, multisignal representation and customization for diverse graph types.

The System Manager offers simple interfaces to access common mobile devices resources (WiFi, 3G, Bluetooth and screen) and builds on the standard Android API [35].

mHealthDroid implements three Service Enablers. The Alerts Enabler provides interfaces to trigger phone calls and text messages. The Notifications Enabler implements text remainders that can be scheduled in a simple way. Moreover, this enabler also provides advanced notifications that can trigger external functionalities or applications. Both Alerts and Notifications Enablers build on the standard Android API. Finally, the Guidelines Enabler provides interfaces to reproduce multimedia content, both locally and remotely stored. The Media Player Android API [36] is used in mHealthDroid to control playback of audio and video files for the local content. For the reproduction of remote multimedia content, the Guidelines Enabler implements a set of functions that build on the YouTube Android Player API [37]. This is particularly practical to access a huge variety of medical and wellbeing content.

The mHealthDroid API is organized into a total of six packages, five of which correspond to each of the aforementioned managers, and an additional one comprising utilities and miscellanea functionalities. The Communication Manager package contains a set of seven classes, respectively devoted to define sensor adapters, data structures and communication protocols. The Storage Manager package is consisted of four main classes, including database adapters, storage interfaces and communication for remote persistence. The Data Processing Manager package contains six classes, one for each individual data processing module, and as for the rest of managers, an additional one for the orchestration of all its modules. The Visualization Manager package consists of two main classes that provide the required functionalities for customized data representation and the control of the canvas. Finally, the System Manager package and Service Enablers package sum up to eleven classes to support the functionalities already described above. All these classes total around 500 methods available to the developer for their use. For a thorough description of the API and its methods, the reader is particularly referred to [21].

## mHealthApp: an exemplary mobile health application

An exemplary application has been developed to illustrate the potential of mHealthDroid. The main features and components of the mHealth Framework have been used during the implementation of this application. As already envisioned during the conception of this framework, the usage of mHealthDroid proved the rapid application development, which in this case was roughly a couple of days. In fact, most of the application development efforts were mainly devoted to the graphical user interface design, whereas the implementation of the core functionalities were easily built on mHealthDroid. The comprehensive set of predefined functions provided by mHealthDroid allowed the mHealthApp developers to easily interface a wearable sensor through simply three lines of code, create a lightweight database system to store the data in just five code lines, or visualize these data on a graph by calling a few predefined functions. This was possible owing to the level of abstraction and programming simplicity introduced by mHealthDroid, also supported with practical templates and helpful documentation provided together with its implementation [21]. The developed mHealthApp is readily available for download from Google Play at [38]. The main features of this application, health data collection, visualization, storage, guidelines and human behavior inference, are described in the following.

### Collection of health data

A key aspect of mHealth applications is the acquisition of people's health data through the use of external wearable devices. mHealthApp provides the functionalities to connect wearable health sensors to a regular mobile device. Shimmer2 sensors [22], supported by the current version of mHealthDroid, are here considered for human health monitoring. These sensors are capable of measuring vital signs and body motion.

The process of adding a new Shimmer device, for the first time, is quite simple. The app user just needs to press a button which initiates the scan of available Bluetooth devices. Then, the pairing of both mobile and wearable sensor devices is performed according to the user selection. This process is not required to be repeated once mobile and wearable device are paired. Gathering and streaming of sensor data to the mobile device can from then on be initiated by the user. After the user presses the corresponding button the app requests the sensor to start collecting data and transfer them to the mobile. The sensors embedded into the mobile device can be also used for human monitoring purposes. Accordingly, mHealthApp provides the user with the possibility of using these sensors similarly to the external ones. All these functionalities essentially build on the mHealthDroid Communication Manager.

### Health data visualization

**Figure 2:**
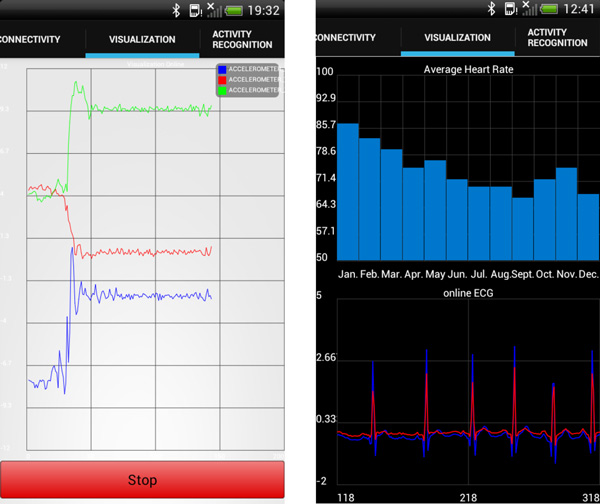
**Examples of representation modes supported by mHealthApp**. (*Left*) Tri- axial acceleration signals are represented at runtime. (*Right*) Monthly average heart rate data is depicted on the top, while continuous 2-leads ECG signals are plotted at the bottom.

The information collected by the wearable or mobile sensors can be depicted through the visualization menu. mHealthApp supports diverse representation modes and plot types. An online representation of the signals is provided to visualize the data collected at runtime, e.g., registered motion data (Figure 2 left and bottom-right). This type of representation is particularly useful for specialists to analyze vital sign patterns. Moreover, average values or general statistics of processed data can be also represented, e.g., heart rate averaged by month (Figure 2 top-right). This kind of graphic is specially devised for the average user, although it is also practical for the expert user.

### Persistent remote storage

The health data collected through the wearable and mobile sensors can be locally stored and uploaded to a remote server. Data transmission procedures, similar to the ones presented for the mHealthDroid implementation, are used here. In mHealthApp, the user simply needs to indicate whether they want to upload the locally stored data and the connection technology, i.e., WiFi or 3G. The user can also select the list of sensor data modalities to be sent remotely.

### Guidelines for health encouragement and personalized recommendations

**Figure 3:**
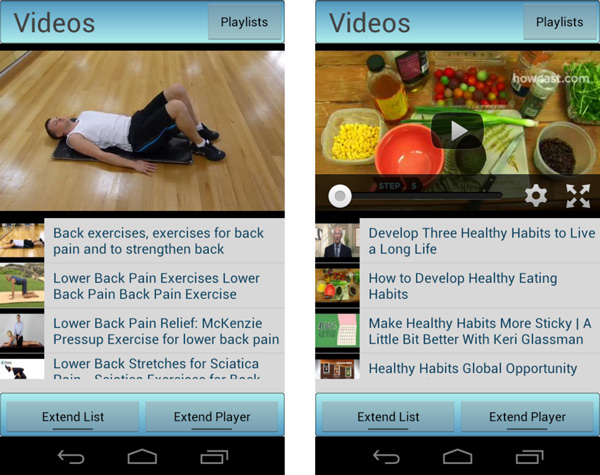
**Examples of video tutorials and guidelines for (*left*) demonstrating rehabilitation exercises and (*right*) encouraging healthy dietary habits**.

mHealth applications are devised to play an important role within the delivery of both traditional and new healthcare services. One of these services corresponds to the empowerment and encouragement of people in their personal health care and wellbeing. mHealthApp comprises an illustrative set of resources to promote education of healthy dietary habits and physical therapy support. The application provides video tutorials and guidelines elaborated by specialists and presented in a categorized and user-friendly fashion (Figure 3). The guidelines may be initiated when the user accesses the corresponding menu or be triggered by personalized notifications. The notifications can be scheduled to a particular date and time by the user.

### Inference of human behavior

**Figure 4:**
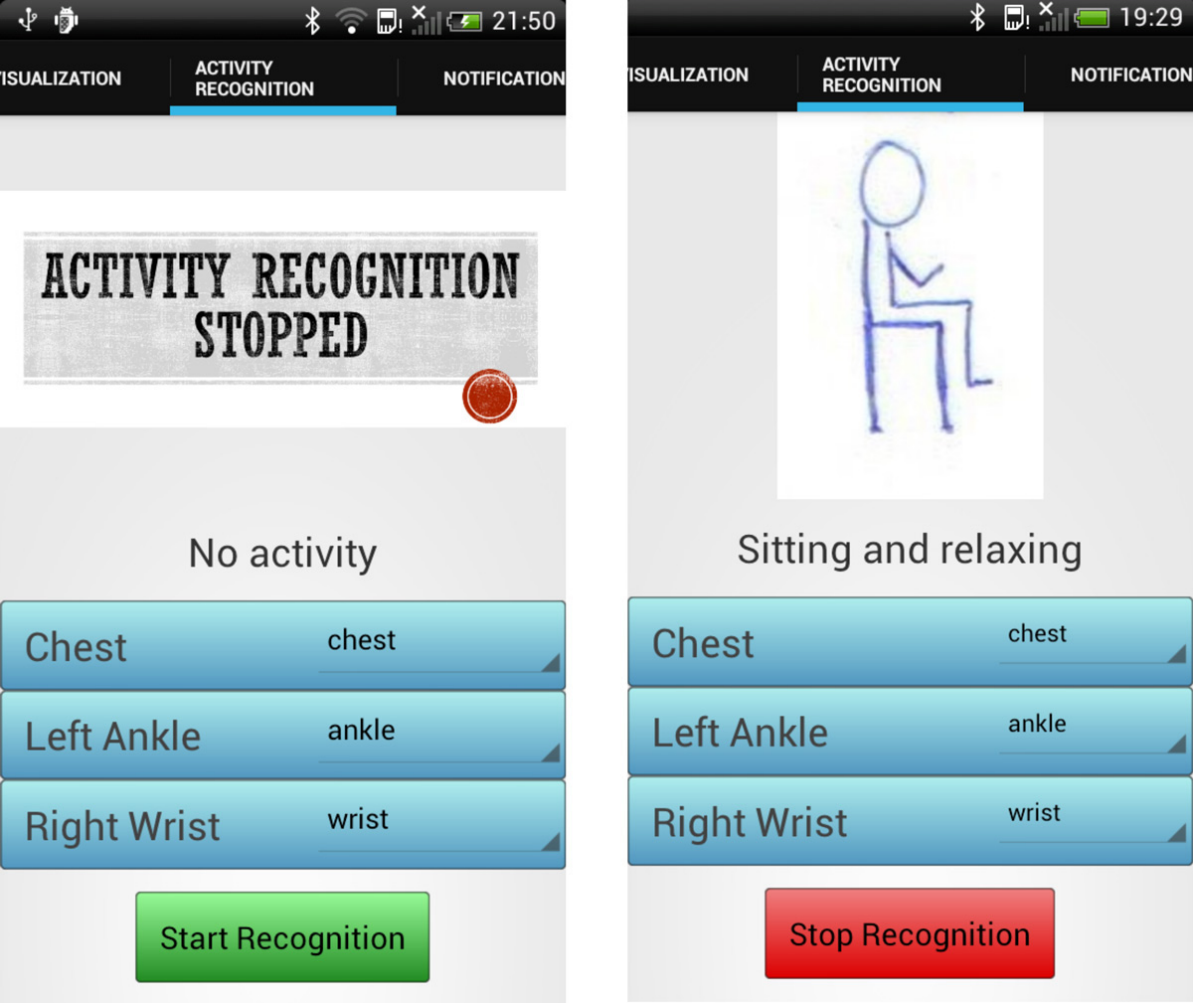
**Snapshots from the activity recognition functionality of mHealthApp: (*left*) the detection process has not been yet initiated by the user; (*right*) the application identifies the activity performed by the user, here, sitting and relaxing**.

mHealthApp provides a means to assess users' daily routines by analyzing the motion data collected through the wearable sensors. The application identifies a set of common activities while the user carries them out (Figure 4). To that end, a human activity recognition model is implemented. This model essentially builds on the mHealthDroid Data Processing Manager. The following section provides an extensive description of the methodology used for the development of the behavior inference functionality.

## Human behavior inference by means of mHealthApp

The analysis of human behavior has attracted very much attention in healthcare, assistance and wellness areas during the recent years. The identification or inference of people's conduct, also known as activity recognition, has been proven of particular interest to promote healthier lifestyles [39,40], prevent unhealthy habits [41,42], detect anomalous behaviors [43-45] or track conditions [46]. Wearable and mobile technologies are extensively used for the inference of human behavior, which makes activity recognition one of the most prominent domains in mHealth.

This section describes a practical solution to the activity recognition problem, which has been developed as part of mHealthApp and aims at illustrating the potential of mHealthDroid. As it is normal practice in the activity recognition domain, a dataset is collected to train and validate the recognition model. The mHealthApp activity recognition model is defined based on prior solutions proved to perform well in similar problems. In order to determine the statistical performance of the recognition model, an offline evaluation is carried out. This type of performance analysis, which is the most recurrently used in activity recognition, consists of training and validating on multiple complementary subsets of the dataset. Once the model is confirmed to perform satisfactorily, it is implemented in mHealthApp by using the mHealthDroid Data Processing Manager functionalities. To prove the correct functioning of mHealthApp during real-time activity recognition, an online evaluation, which is less frequent in activity recognition studies, is performed.

### MHEALTH dataset

**Figure 5:**
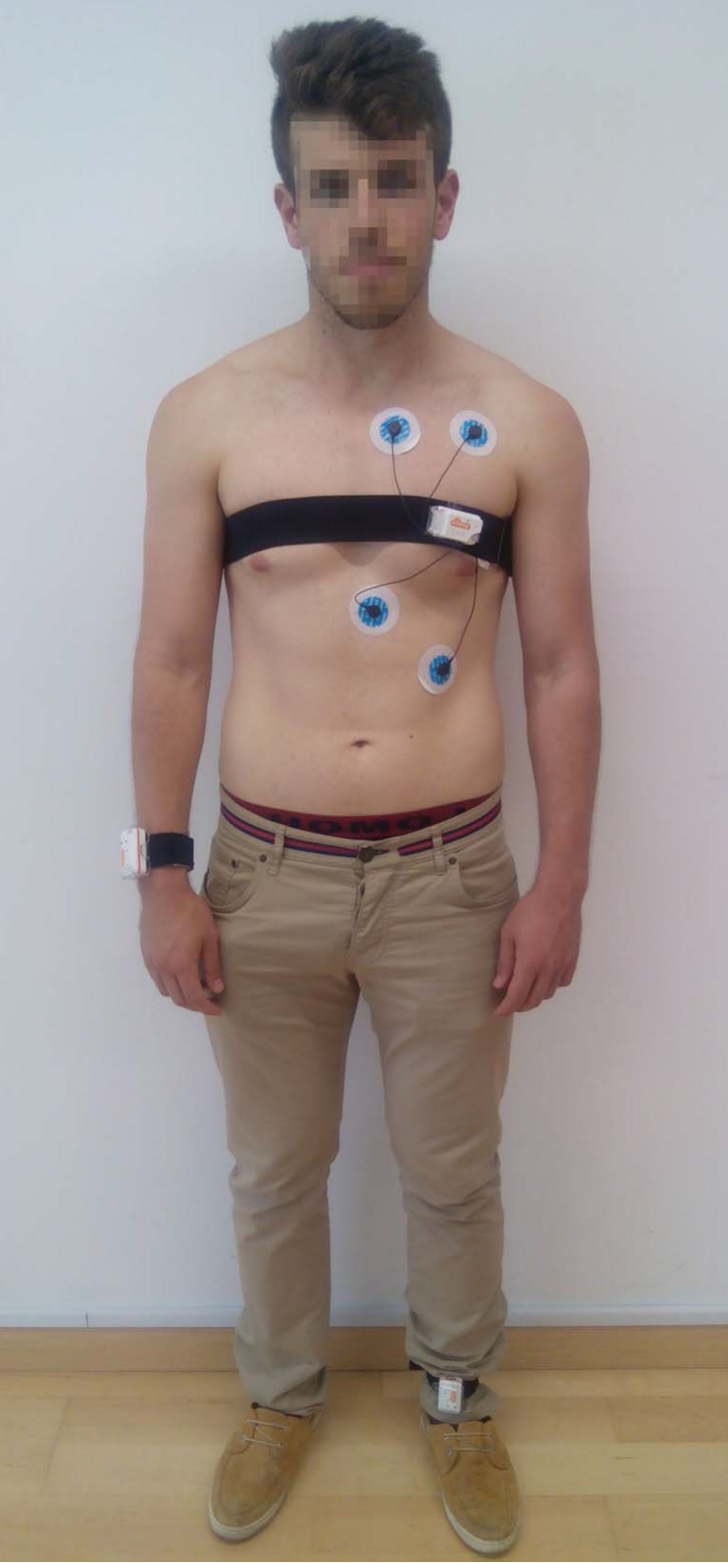
**Study setup and sensor deployment**.

The collected dataset, hereafter MHEALTH dataset, comprises body motion and vital signs recordings, for ten volunteers of diverse profile, while performing 12 physical activities (Table 1). Shimmer2 wearable sensors [22] were used for the recordings. The sensors were respectively placed on the subject's chest, right wrist and left ankle and attached by using elastic straps as illustrated in Figure 5. The use of multiple sensors permits us to measure the motion experienced by diverse body parts, namely, the acceleration, the rate of turn and the magnetic field orientation, thus better capturing the body dynamics. The sensor positioned on the chest also provides 2-lead ECG measurements, which are not used in this work for the development of the recognition model, but rather collected for future work purposes. This information can be used, for example, for basic heart monitoring, checking for various arrhythmias or looking at the effects of exercise on the ECG. The sampling rate used for all sensing modalities is of 50 Hz, which is considered sufficient for capturing human activity. All sessions were recorded using a video camera. The video recording was used to label the data and to check anomalous or unexpected patterns in the signals.

**Table 1:** Activity set.

Activity Set	
L1: Standing still (1 min)	L7: Frontal elevation of arms (20*×*)
L2: Sitting and relaxing (1 min)	L8: Knees bending (crouching) (20*×*)
L3: Lying down (1 min)	L9: Cycling (1 min)
L4: Walking (1 min)	L10: Jogging (1 min)
L5: Climbing/descending stairs (1 min)	L11: Running (1 min)
L6: Waist bends forward (20*×*)	L12: Jump front & back (20*×*)

In brackets are the number of repetitions (N*×*) or the duration of the exercises (min).

This dataset is found to generalize to common activities of daily living, given the diversity of body parts involved in each one (e.g., frontal elevation of arms *vs*. knees bending), the intensity of the actions (e.g., cycling *vs*. sitting and relaxing) and their execution speed or dynamicity (e.g., running *vs*. standing still). The activities were collected in an out-of-lab environment, with no constraints on the way they had to be executed. In any case, the subjects were asked to try their best when executing them. The MHEALTH dataset is readily available for download at [47].

### mHealthApp activity recognition model

The activity recognition process consists of a set of steps, already introduced during the description of the Data Processing Manager, that mainly combine signal processing, pattern recognition and machine learning techniques to define a specific activity recognition model [48]. In the following, the activity recognition model used in mHealthApp is described.

The motion signals provided by each of the three wearable devices are used for the activity identification. Concretely, the triaxial acceleration data is considered, since this is the most prevalent sensor modality in previous activity recognition approaches [49,50]. No preprocessing of the data is applied to avoid the removal of relevant information. A non-overlapping sliding window approach is considered for the segmentation process. This technique is supported by the current version of the mHealthDroid Segmentation module. The window size is particularly set to two seconds, since it proves to provide a good trade-off between recognition speed and accuracy for the activities of interest [51]. Mean, standard deviation, maximum and minimum are selected from the set of features available in the mHealthDroid Feature Extraction module. These features are typically used in activity recognition for their discrimination potential and easy interpretation in the acceleration domain [52-57]. Decision trees are used for the classification process, which have been shown to perform well in combination with similar features and activities [52,58,59]. Particularly, the J48 decision tree model implemented by the mHealthDroid Classification module is used here.

### Offline evaluation of the mHealthApp activity recognition model

The offline evaluation of the activity recognition model is performed on the MHEALTH dataset and through a cross-validation process. The cross-validation technique involves partitioning the dataset into complementary subsets, performing the analysis on one subset (training set) and validating the analysis on the other subset (validation or testing set). Although there exist several cross-validation approaches, leave-one-subject-out cross validation and ten-fold cross-validation are the most widely used in activity recognition. In this study the latter validation technique is particularly considered since it leads to a better estimate of the performance of the recognition model [60]. Moreover, the ten-fold cross-validation process is repeated 100 times to ensure statistical robustness and to procure an asymptotic convergence to a correct estimation of the system performance [61].

Diverse metrics may be used to evaluate the performance of the recognition system. The confusion matrix stands out among others, since it summarizes in a single matrix all the information corresponding to the performance evaluation. From this matrix, several other metrics can be simply derived such as the sensitivity (SE), specificity (SP), positive predictive value (PPV), negative predictive value (NPV) and F-score. An extensive review of these and other metrics can be seen in [62].

**Figure 6:**
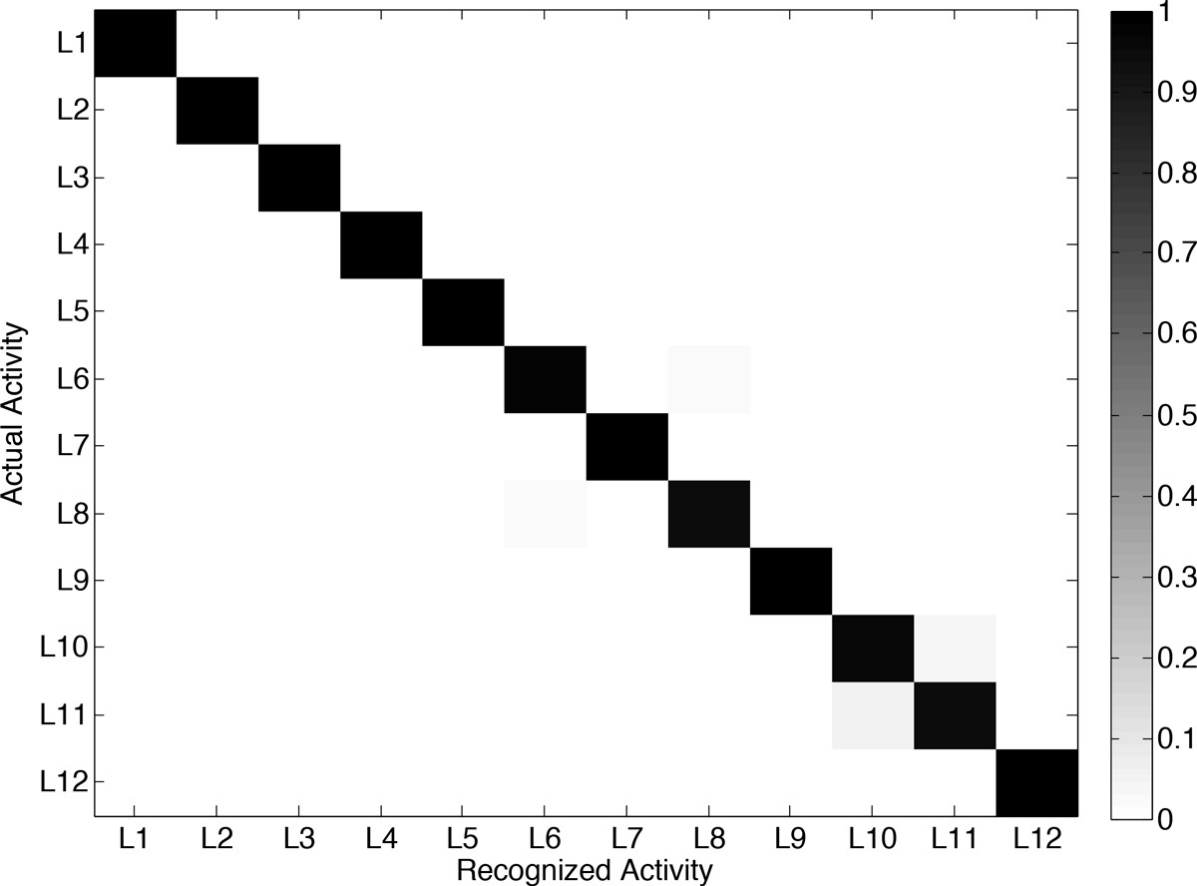
**Confusion matrix obtained from the offline evaluation of the activity recognition model**. Activities are identified through the labels introduced in Table 1.

The results obtained after evaluation are shown in Figure 6 and Table 2. As it can be observed, the developed system provides very promising recognition capabilities for the considered activities. The confusion matrix (Figure 6) is practically diagonal, which represents a performance close to absolute. This is further confirmed for each particular activity, considering the values obtained for each performance metric (Table 2). In fact, daily activities such as standing still, sitting, lying down or walking are unequivocally identified. Subtle misclassifications are seen for other activities that are more dependent on the physical conditions of the subjects, such as running or knees bending. At any rate, from the successful outcomes of this evaluation it can be concluded that the proposed system is a promising activity recognizer.

**Table 2:** Recognition performance for each activity class for the offline evaluation.

Activity	SE	SP	PPV	NPV	F-score
L1	1.00	1.00	1.00	1.00	1.00
L2	1.00	1.00	1.00	1.00	1.00
L3	1.00	1.00	1.00	1.00	1.00
L4	1.00	1.00	1.00	1.00	1.00
L5	0.99	1.00	0.99	1.00	0.99
L6	0.97	1.00	0.97	1.00	0.97
L7	1.00	1.00	0.99	1.00	0.99
L8	0.95	1.00	0.97	1.00	0.96
L9	1.00	1.00	0.99	1.00	1.00
L10	0.96	0.99	0.94	1.00	0.95
L11	0.94	1.00	0.96	0.99	0.95
L12	0.99	1.00	0.99	1.00	0.99

Each metric respectively correspond to the sensitivity (SE), specificity (SP), positive predictive value (PPV), negative predictive value (NPV) and F-score.

### Online evaluation of the mHealthApp activity recognition model

The online validation allows us to corroborate the potential of the mHealthApp activity recognition model at runtime. The recognition model implemented in mHealthApp builds on the complete MHEALTH dataset. The validation is performed on a different set of subjects to the one considered for the model training. A total of five volunteers were asked to perform the complete activity set (Table 1). Each activity was carried out during 30 seconds as it was considered to be enough given the characteristics of the exercises. The activities were performed in various outdoor scenarios for the users' convenience and to reduce their awareness as much as possible. During the executions both user's activity and smartphone's screen (Figure 4) were recorded on video for the evaluation of the system performance. This was considered to be a more tractable approach than using commentary sheets for the activities annotation. The performance is evaluated by comparing both actual and detected activities based on the observation of the video recordings. Actual and predicted activities are aligned taking into account the 1-second delay associated to the data segmentation process. Transitions among the activities of interest are left out of the study since a null-class rejection schema has not been explicitly implemented [63].

**Figure 7:**
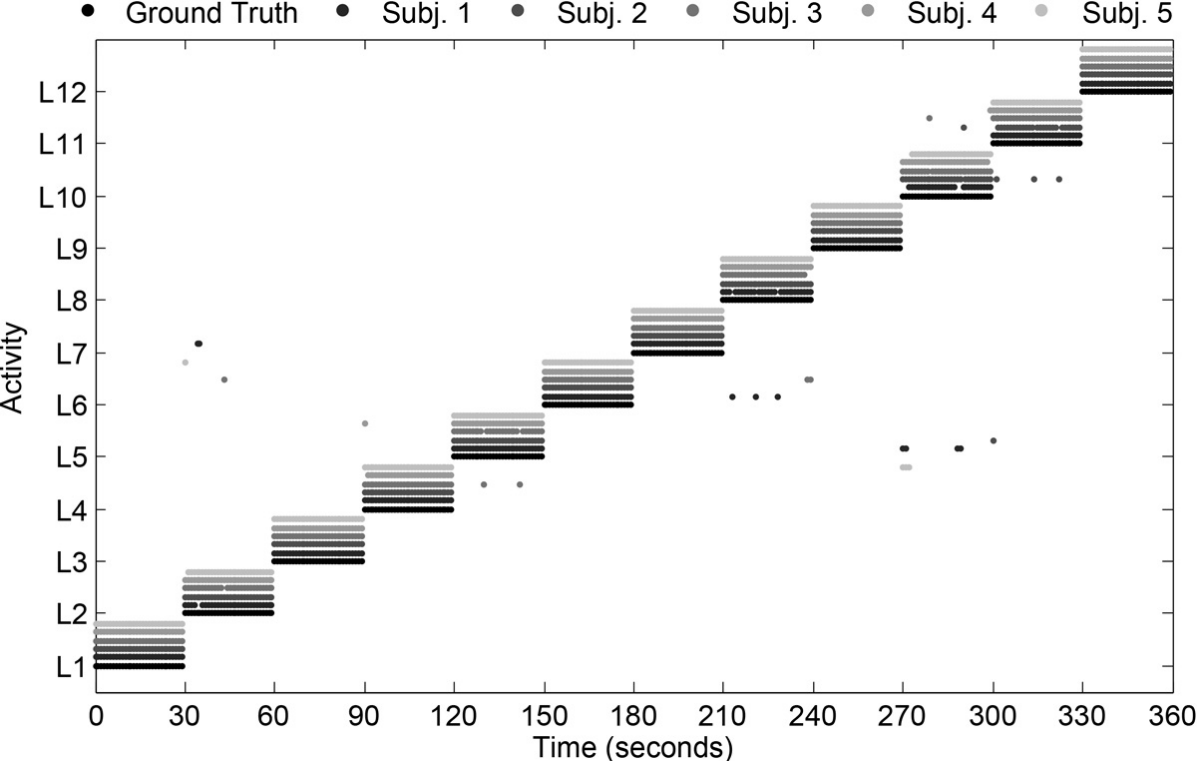
**Activities detected by the proposed recognizer during online evaluation of the system and for various subjects**. The actual activities are represented by the ground-truth labels. Activities are identified through the labels introduced in Table 1.

**Figure 8:**
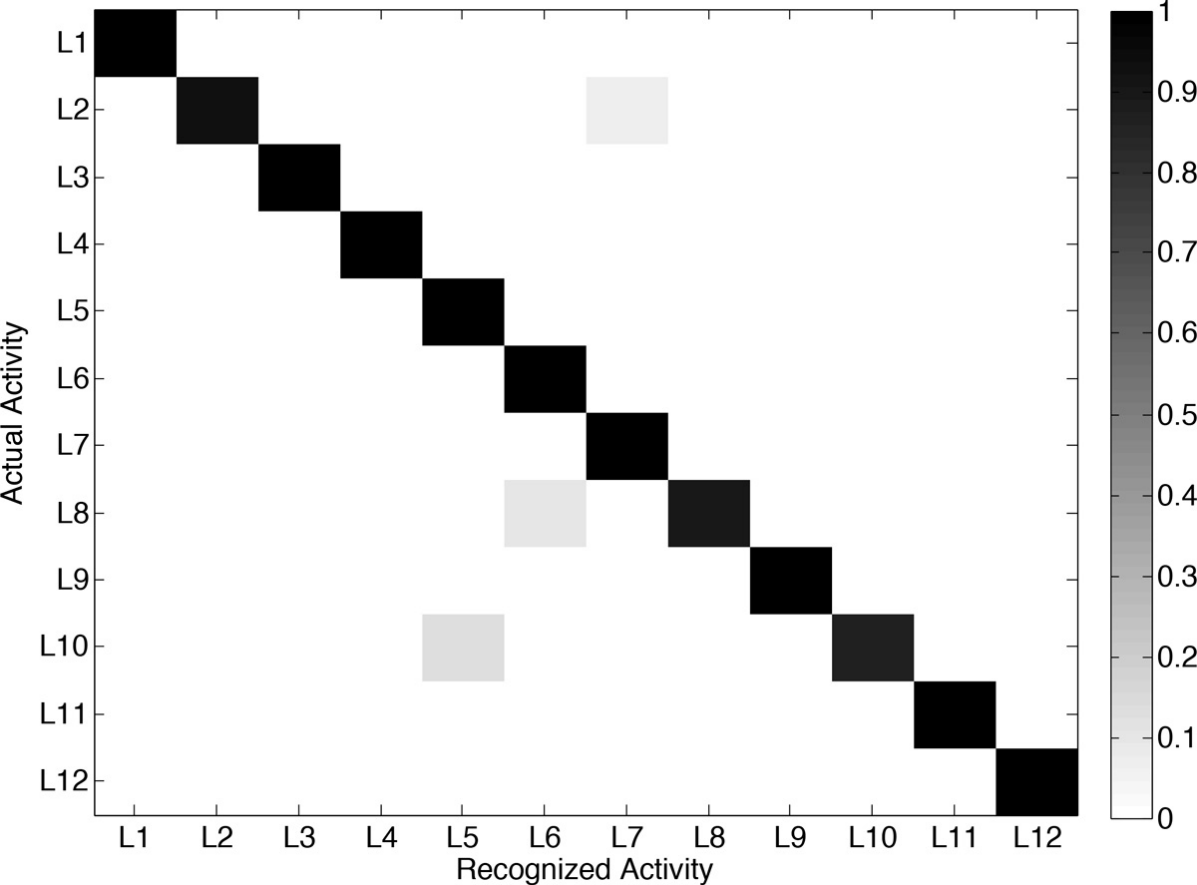
**Confusion matrix obtained from the online evaluation of the activity recognition model**. Activities are identified through the labels used in Table 1.

The activities detected by mHealthApp, for each user, are depicted in Figure 7. In broad strokes, it can be said that the system shows good recognition capabilities. Only a few outliers or misclassifications are observed. For example, during the identification of "sitting and relaxing", the model sometimes interprets that the users are bending their waist forward or elevating their arms. This is explained by some abrupt movements observed during the execution of this activity for some of the participants. Similarly, some errors are found for the detection of "knees bending or crouching", which is confused here again with "waist bend forwards". This is a consequence of some difficulties encountered by part of the users while performing this exercise, which translated into a moderate sway back and forth. Finally, a few misclassifications are observed among "walking", "jogging" and "running", which are basically originated from the varying cadence with which these activities were executed by the subjects. All these conclusions are further supported by the confusion matrix (Figure 8) and the individual performance metrics for each activity (Table 3). In summary, the developed mHealthApp activity recognition model is not only shown to operate in an experimental basis but also proved to work well during the normal use of the system.

**Table 3:** Recognition performance for each activity class for the online evaluation.

Activity	SE	SP	PPV	NPV	F-score
L1	1.00	1.00	1.00	1.00	1.00
L2	0.93	1.00	1.00	0.99	0.97
L3	1.00	1.00	1.00	1.00	1.00
L4	1.00	1.00	1.00	1.00	1.00
L5	1.00	0.99	0.88	1.00	0.94
L6	1.00	0.99	0.91	1.00	0.95
L7	1.00	0.99	0.94	1.00	0.97
L8	0.90	1.00	1.00	0.99	0.95
L9	1.00	1.00	1.00	1.00	1.00
L10	0.87	1.00	1.00	0.99	0.93
L11	1.00	1.00	1.00	1.00	1.00
L12	1.00	1.00	1.00	1.00	1.00

Each metric respectively correspond to the specificity (SP), sensitivity (SE), positive predictive value (PPV), negative predictive value (NPV) and F-score.

## Conclusions

A novel mHealth framework intended to facilitate the development of mobile health applications in a simple and agile fashion has been presented in this paper. The framework has been designed taking into account crucial requirements of mHealth technologies and applications. This work has also introduced mHealthDroid, an open source implementation of the proposed mHealth Framework that operates on the Android OS. This implementation aims at bringing together mobile devices and heterogeneous multimodal sensors including both research and commercial systems. mHealthDroid supports basic and advanced features of mHealth applications such as resource and communication abstraction, biomedical data acquisition, health knowledge extraction, persistent data storage, adaptive visualization, system management and value-added services such as intelligent alerts, recommendations and guidelines.

An exemplary app has also been provided along with this work to showcase the potential of mHealthDroid. mHealthApp implements mechanisms for collection and visualization of health data, persistent remote storage and personalized health and wellbeing guidelines. This app also provides an advanced means to detect and track human behavior, a functionality that has been further validated in this work through extensive experimentation. The agility and simplicity gained by using the mHealthDroid API is proved through the reduced time required for developing an app of these characteristics.

Ultimately, mHealthDroid aims at bringing together developers, healthcare professionals, academics and health enthusiasts to exchange ideas and cooperate in the definition of valuable tools for a healthier world. Accordingly, the authors encourage the community to contribute to this innovative platform by supporting the use of the latest sensors, incorporating new behavioral algorithms or simply making use of it for the development of novel mobile health applications.

## Consent

Written informed consent was obtained from the participants for publication of this case report and any accompanying images. A copy of the written consent is available for review by the Editor of this journal.

## Competing interests

The authors declare that they have no competing interests.

## Authors' contributions

OB was the lead researcher of this work, designed the framework and wrote the paper together with CV. RG and AS implemented the initial version of mHealthDroid and the app described in this work. JAH and SL provided guidance for the framework definition. MD, HP and IR reviewed the manuscript for scientific content. All authors read and approved the final manuscript.

## Declarations

Publication costs for this article were funded by the Regional Government of Andalusia under grant agreement P12-TIC 2082.

This article has been published as part of *BioMedical Engineering OnLine *Volume 14 Supplement 2, 2015: Select articles from the 2nd International Work-Conference on Bioinformatics and Biomedical Engineering (IWBBIO 2014). The full contents of the supplement are available online at http://www.biomedical-engineering-online.com/supplements/14/S2.
